# Uncovering electrophysiological and vascular signatures of implicit emotional prosody

**DOI:** 10.1038/s41598-020-62761-x

**Published:** 2020-04-02

**Authors:** Sarah Steber, Nicola König, Franziska Stephan, Sonja Rossi

**Affiliations:** 1 ICONE - Innsbruck Cognitive Neuroscience, Department for Hearing, Speech, and Voice Disorders, Medical University of Innsbruck, 6020 Innsbruck, Austria; 2 Department of Psychology, University of Innsbruck, 6020 Innsbruck, Austria; 3 Department of Educational Psychology, Faculty of Education, University of Leipzig, 04109 Leipzig, Germany

**Keywords:** Cognitive neuroscience, Emotion

## Abstract

The capability of differentiating between various emotional states in speech displays a crucial prerequisite for successful social interactions. The aim of the present study was to investigate neural processes underlying this differentiating ability by applying a simultaneous neuroscientific approach in order to gain both electrophysiological (via electroencephalography, EEG) and vascular (via functional near-infrared-spectroscopy, fNIRS) responses. Pseudowords conforming to angry, happy, and neutral prosody were presented acoustically to participants using a passive listening paradigm in order to capture implicit mechanisms of emotional prosody processing. Event-related brain potentials (ERPs) revealed a larger P200 and an increased late positive potential (LPP) for happy prosody as well as larger negativities for angry and neutral prosody compared to happy prosody around 500 ms. FNIRS results showed increased activations for angry prosody at right fronto-temporal areas. Correlation between negativity in the EEG and activation in fNIRS for angry prosody suggests analogous underlying processes resembling a negativity bias. Overall, results indicate that mechanisms of emotional and phonological encoding (P200), emotional evaluation (increased negativities) as well as emotional arousal and relevance (LPP) are present during implicit processing of emotional prosody.

## Introduction

For a successful interpersonal communication, correct identification and processing of emotional states of one’s counterpart are necessary. Emotions can be transported via multiple modalities like facial expressions and hand gestures but also via prosody of speech. Prosody, that is, the melodic contour of a word or sentence, originates from the interaction of pitch, loudness, rhythm, intensity, and frequency of specific verbalizations^1^. The terms emotional or affective prosody refer to those intonational patterns carrying emotional states such as a happy or angry emotion^2,3^. While research on emotional processing in the visual domain (e.g., facial expressions, pictures with emotional content) already been intensively conducted, emotional prosody processing is still less investigated. Identifying emotions through the voice is highly relevant in everyday life, even more so when visual information is not available, as acoustic parameters have the ability to travel longer distances, while visual cues are in need of close proximity to the target^4^. It is known that with a combination of several modalities delivering emotional information, emotion identification improves, however, when contrasting unimodal information, emotions seem to be easier recognizable from speech than faces^5,6^. Furthermore, the emotional auditory input is able to direct attention to relevant emotional stimuli in the environment and delivers additional crucial information for how to react to facial expressions (e.g., recognition facilitation of visual cues)^7–9^.

Neuroscientific studies bear the potential to provide deeper understanding of mechanisms underlying emotional identification processes beyond the pure behavioral level. Therefore, we opted for a multi-methodological approach of two sensitive neuroscientific methods to provide important insights in both temporal and spatial mechanisms during the processing of emotional prosodic cues. Electrophysiological and vascular responses were assessed simultaneously by electroencephalography (EEG), specifically the analysis of event-related brain potentials (ERPs), and functional near-infrared spectroscopy (fNIRS). Both methods have proven to be very beneficial for the investigation of acoustic stimuli, as they are both soundless, do not interfere with each other, and provide an ecologically valid setting^10^. As previous studies on emotional prosody mostly used real words^11–14^, sentences^15–18^, and specific tasks or additional visual aids^16,17,19,20^, we aimed at investigating implicit emotional processing by adopting a passive listening paradigm using pseudowords unfamiliar to the subjects. Real words that carry a semantic content per se might confound emotional processing, at least at the individual subject level. Similarly, a sentential context can impact emotional processing from a semantic point of view. Furthermore, specific tasks could cover up pure emotional identification processes by directing attention to specific cues or might at least alter neural activation patterns^21,22^.

While segmental linguistic features like phonology and syntax mainly recruit left-hemispheric brain regions, suprasegmental features like prosody are assumed to be predominantly supported by the right hemisphere (e.g., the Dynamic Dual Pathway Model^23^ or the Dual Stream Model of Speech processing^24,25^). Regarding emotional processing, the right-hemisphere hypothesis of emotion (RHH) suggests a predominantly right-hemispheric recruitment of brain regions for emotional stimuli as well^26^, and research indicates that both linguistic and affective prosody seem to share the same neurological underpinnings^27^. Acoustic emotional stimuli are often characterized by an exaggerated prosodic contour, which might explain this corresponding right-hemispheric lateralization. Another model, the valence-arousal hypothesis (VH), assigns the processing of different emotions to different hemispheres. The VH suggests that negative emotions are accompanied by more right-sided anterior activity, while pleasant emotions are associated with more left-sided anterior activity^28–30^. Support for both RHH and VH was found by several studies (please refer to^31^ for an overview), however, an overall right-hemispheric dominance for emotional (prosody) processing has again been proposed in recent reviews^32,33^.

FNIRS literature on emotional prosody processing in adults is very scarce. Despite providing a good spatial resolution of cortical activations, the fNIRS does not reach deeper brain regions like the amygdala, which is known to be predominantly involved in emotional processing in general^34,35^ but also in emotional prosody processing in particular^36^. However, in contrast to functional magnetic resonance imaging (fMRI), fNIRS as a silent optical imaging method provides a very beneficial setting for the investigation of acoustic emotional stimuli^10^. Frühholz, Trost, and Kotz^33^ propose a network model of emotional processing in the auditory domain, comprising the limbic system, the auditory cortex (AC), the superior temporal cortex (STC), insula, orbital frontal cortex (OFC), inferior frontal cortex (IFC), and medial frontal cortex (MFC). Authors suggest that emotional information is fed from the STC to the IFC for a first cognitive evaluation of the emotional content while information decoded by the amygdala implicitly without attentional focus is sent to the MFC where additional emotional appraisal, evaluation and regulation processes take place.

Several fMRI studies on emotional prosody using different materials (e.g., sentences^37^, adjectives^13^, and word-like utterances^38^) and tasks revealed a quite consistent pattern of activations mainly in right fronto-temporal brain regions for emotional compared to neutral stimuli. Using 4 pseudowords from the Geneva Multimodal Emotion Portrayal database^39^, one fMRI study contrasting neutral and angry prosody showed activations for angry prosody in the right posterior STG and bilateral IFG subregions during an implicit listening task^19^.

One fNIRS study by Plichta *et al*.^40^ revealed that both pleasant and unpleasant sounds compared to neutral sounds lead to an increased activation in the AC. This effect was more pronounced in the right hemisphere. In addition, an fNIRS study^41^ found an increased right-hemispheric activation of the STG when listening to emotional (frightened, disgusted) compared to neutral sounds. To our knowledge, only one fNIRS study specifically investigated emotional processing in speech instead of mere sounds. Zhang, Zhou, and Yuan^42^ found increased activations in the STC for emotional compared to neutral mandarin pseudosentences during a passive listening task with increased right-hemispheric involvement during emotional prosody perception.

As shown here, interestingly, most research using imaging techniques in the context of emotional prosody finds differences between emotional and neutral stimuli but fails to reveal differences in activation between positive and negative intonation patterns^42^.

With respect to electrophysiological evidence on prosody processing, literature has found effects at both early and later processing stages. Modulations in N100 related to pitch and loudness characteristics of stimuli as well as P200 components reflecting an initial process of emotional encoding and arousal have been reported^43^. The P200 often is followed by a long-lasting late positive potential (LPP) with a maximum between 300 and 1000 ms after stimulus onset indicating extensive emotional processing mechanisms. The LPP has been found in both the visual and the auditory domain, is usually distributed on centro-parietal electrodes, and more pronounced when emotional arousal or motivational relevance of the respective stimulus is higher^15,44–47^. With respect to emotional arousal, direction of effects in P200 components can predict LPP modulations^14,43^.

Electrophysiological studies using pseudowords in the context of emotional prosody are scarce. Using pseudosentences, however, ERP studies^15,48^ found increased LPPs for emotional compared to neutral prosody. One study^49^ with pseudowords (angry vs. sad prosody) also found enhanced positivities for the emotional prosody (i.e., emotion identification) but not for the linguistic prosody (i.e., word syllable stress identification) task demands in a dichotic listening paradigm. However, authors did not compare emotional prosody to neutral prosody.

To our knowledge, so far, no other neuroscientific study investigated implicit processing of emotional prosody (angry vs. happy vs. neutral prosody) in single pseudowords completely different from the subjects’ native language in order to exclude a potential linguistic influence. Pseudowords in the present study conformed to foreign phonotactic rules (i.e., the combination of different phonemes^50^). Based on previous research, we expect to find increased right-hemispheric activations for emotional compared to neutral prosody in the fNIRS, presumably in regions comprising STC, MFC and IFC. Furthermore, we expect to find larger LPPs for emotional compared to neutral prosody in the EEG. As brain activation patterns might change due to task variations, investigating implicit processing with participants listening passively to the speech input without performing any specific task might also bear the potential to shed light on differences not only between neutral and emotional stimuli but also between different emotional categories (e.g., angry vs. happy). Furthermore, simultaneously applying two neuroscientific methods allows correlating electrophysiological and vascular responses of corresponding EEG and fNIRS findings in order to uncover mechanisms equivalent in nature. Similar approaches were successfully adopted in EEG-fNIRS^51,52^ as well as EEG-fMRI studies^53^.

## Results

### EEG results

**Figure 1:**
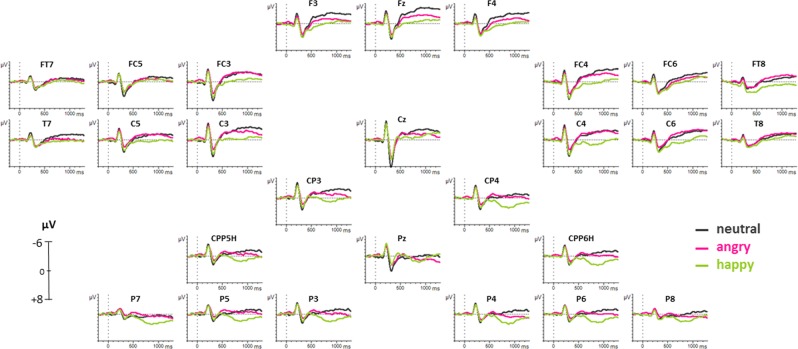
ERP results on all electrodes for neutral, angry, and happy pseudowords. Negative polarity is plotted upwards. An 8 Hz low-pass filter was applied for presentation purposes only.

**Figure 2:**
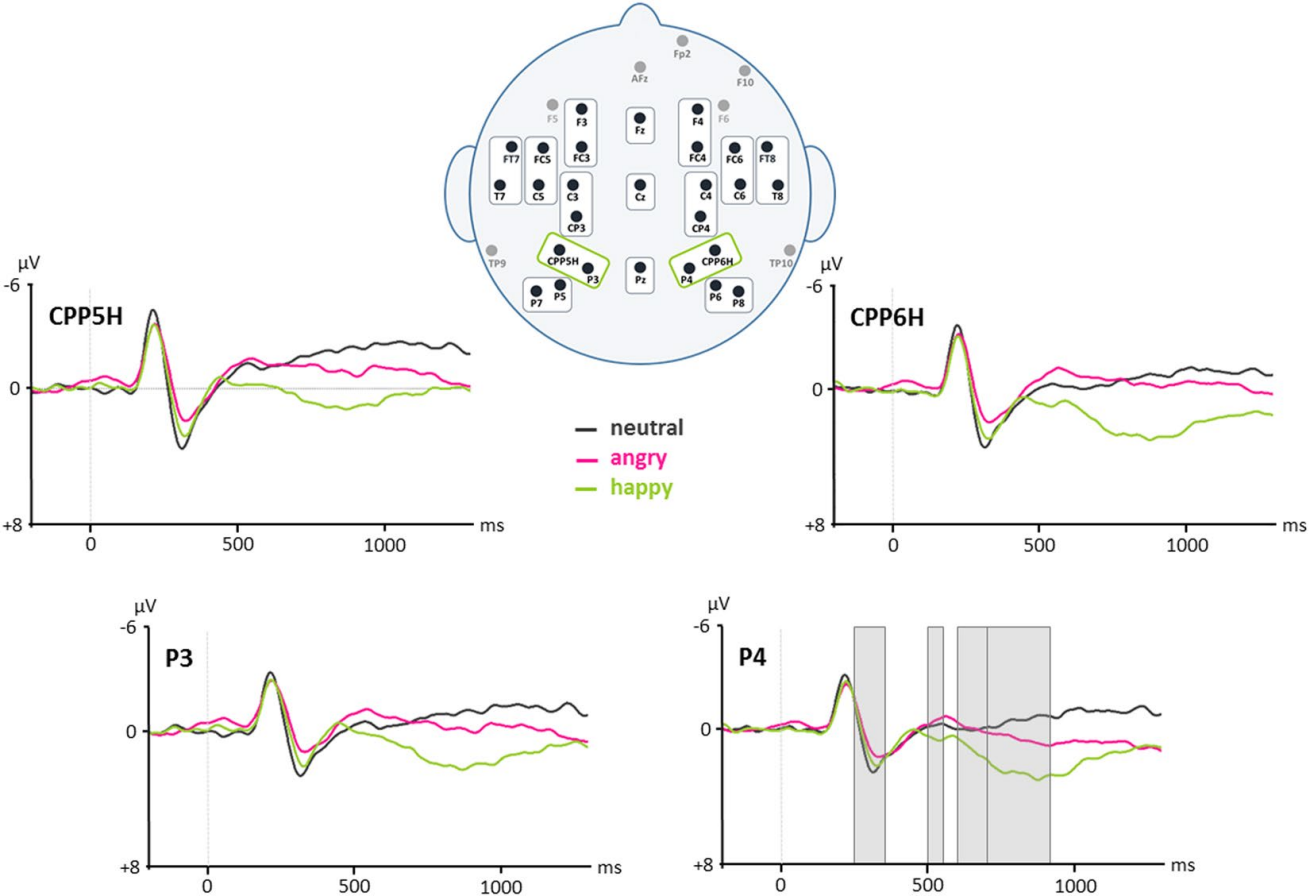
ERP results on left and right centro-parietal superior ROIs (CPP5H/P3, CPP6H/P4) for neutral, angry, and happy pseudowords. Negative polarity is plotted upwards. An 8 Hz low-pass filter was applied for presentation purposes only. Analyzed time windows that revealed significant results are indicated on electrode P4 (250–350 ms, 500–550 ms, 600–700 ms, 700–900 ms).

On all time windows, a four-way ANOVA (Condition × Halves × Hemisphere × Region) was performed. Results of these ANOVAs and direction of effects are provided in the text as well as in Figs. 1 and 2, for a detailed overview of statistical results of post-hoc *t*-tests, please refer to Table 1.

For time window 100–150 ms, no main or interaction effect reached significance, neither on lateral ROIs nor on midline electrodes.

**Table 1:** Summary of EEG results with all significant post-hoc *t*-tests.

time window	ROI/electrode	df	*t*	*p*	effect
250–350 ms	frontal	47	−2.252	0.029	happy > angry (+)
	centro-parietal	47	−2.429	0.019	
	frontal	47	−2.711	0.009*	neutral > angry (+)
	fronto-temporal superior	47	−3.033	0.004*	
	centro-parietal	47	−4.505	<0.001*	
	parietal superior	47	−3.823	<0.001*	
	parietal inferior	47	−3.169	0.003*	
	Fz	47	−2.633	0.011*	
	Cz	47	−4.589	<0.001*	
	Pz	47	−3.872	<0.001*	
	Cz	47	−3.05	0.004*	neutral > happy (+)
	Pz	47	−2.963	0.005*	
500–550 ms	frontal	47	−2.924	0.005*	angry > happy (−)
	centro-parietal	47	−2.517	0.015	
	parietal superior	47	−2.011	0.05	
	Fz	47	−2.075	0.044	
	Cz	47	−2.151	0.037	
	frontal	47	4.76	<0.001*	neutral > happy (−)
	fronto-temporal superior	47	2.219	0.031	
	fronto-temporal inferior	47	2.207	0.032	
	centro-parietal	47	3.166	0.003*	
	Fz	47	4.576	<0.001*	neutral > angry (−)
600–700 ms	all lateral ROIs	47	−2.162	0.036	happy > angry (+)
	all lateral ROIs	47	2.296	0.026	happy > neutral (+)
	Fz	47	4.102	<0.001*	happy > neutral (+)
		47	2.456	0.018	angry > neutral (+)
700–900 ms	frontal	47	−2.758	0.008*	happy > angry (+)
	centro-parietal	47	−2.885	0.006*	
	parietal superior	47	−2.608	0.012*	
	parietal inferior	47	−2.602	0.012*	
	frontal	47	3.129	0.003*	happy > neutral (+)
	centro-parietal	47	3.767	<0.001*	
	parietal superior	47	3.514	0.001*	
	parietal inferior	47	3.404	0.001*	
	midline electrodes	47	2.253	0.029	happy > neutral (+)
		47	2.215	0.032*	angry > neutral (+)

Significant effects (i.e., increased positivities (+) and increased negativities (−)) were found on frontal (F3, FC3, F4, FC4), fronto-temporal superior (FC5, C5, FC6, C6), fronto-temporal inferior (FT7, T7, FT8, T8), centro-parietal (C3, CP3, C4, CP4), parietal superior (CPP5H, P3, CPP6H, P4), and parietal inferior (P7, P5, P8, P6) lateral ROIs as well as on midline electrodes (Fz, Cz, Pz). *P*-values that survived FDR correction are marked with an asterisk.

For time window 250–350 ms, the ANOVA revealed a significant main effect of Condition [*F*(2,94) = 5.772, *p* = 0.008] and a significant interaction of Condition × Region [*F*(10,470) = 2.198, *p* = 0.049] on lateral ROIs, as well as a significant main effect of Condition [*F*(2,94) = 8.143, *p* = 0.001] and an interaction effect of Condition × Electrode [*F*(4,188) = 8.301, *p* < 0.001] on midline electrodes. Post-hoc *t*-tests showed tendentially larger positivities for happy than angry prosody. Furthermore, larger positivities for neutral than angry prosody as well as for neutral than happy prosody were found.

On time window 500–550 ms, the ANOVA showed a significant main effect of Condition [*F*(2,94) = 4.852, *p* = 0.015] and an interaction effect of Condition × Region [*F*(10,470) = 4.768, *p* < 0.001] on lateral ROIs as well as an interaction effect of Condition × Electrode [*F*(4,188) = 10.786, *p* < 0.001] on midline electrodes. Post-hoc *t*-tests revealed tendentially larger negativities for angry than happy prosody as well as larger negativities for neutral than happy prosody. One additional effect was found on midline electrode Fz only, with an increased negativity for neutral compared to angry prosody.

On time window 600–700 ms, the ANOVA showed a significant main effect of Condition [*F*(2,94) = 3.966, *p* = 0.030] on lateral ROIs as well as an interaction effect of Condition × Electrode [*F*(4,188) = 6.409, *p* = 0.003] on midline electrodes. Post-hoc *t*-tests revealed a tendency of increased positivities for happy prosody compared to both angry as well as neutral prosody. On midline electrode Fz only, *t*-tests showed increased positivities for happy and a tendency of increased positivities for angry prosody compared to neutral prosody.

On time window 700–900 ms, the ANOVA showed a significant main effect of Condition [*F*(2,94) = 7.019, *p* = 0.004] and an interaction effect of Condition × Region [*F*(10,470) = 2.982, *p* < 0.012] on lateral ROIs as well as a main effect of Condition [*F*(4,188) = 3.957, *p* = 0.022] on midline electrodes. On lateral regions, post-hoc *t*-tests showed larger positivities for happy than angry prosody as well as increased positivities for happy than neutral prosody. On midline electrodes, *t*-tests revealed larger positivities for angry as well as a tendency of increased positivities for happy prosody compared to neutral prosody.

### fNIRS results

**Figure 3:**
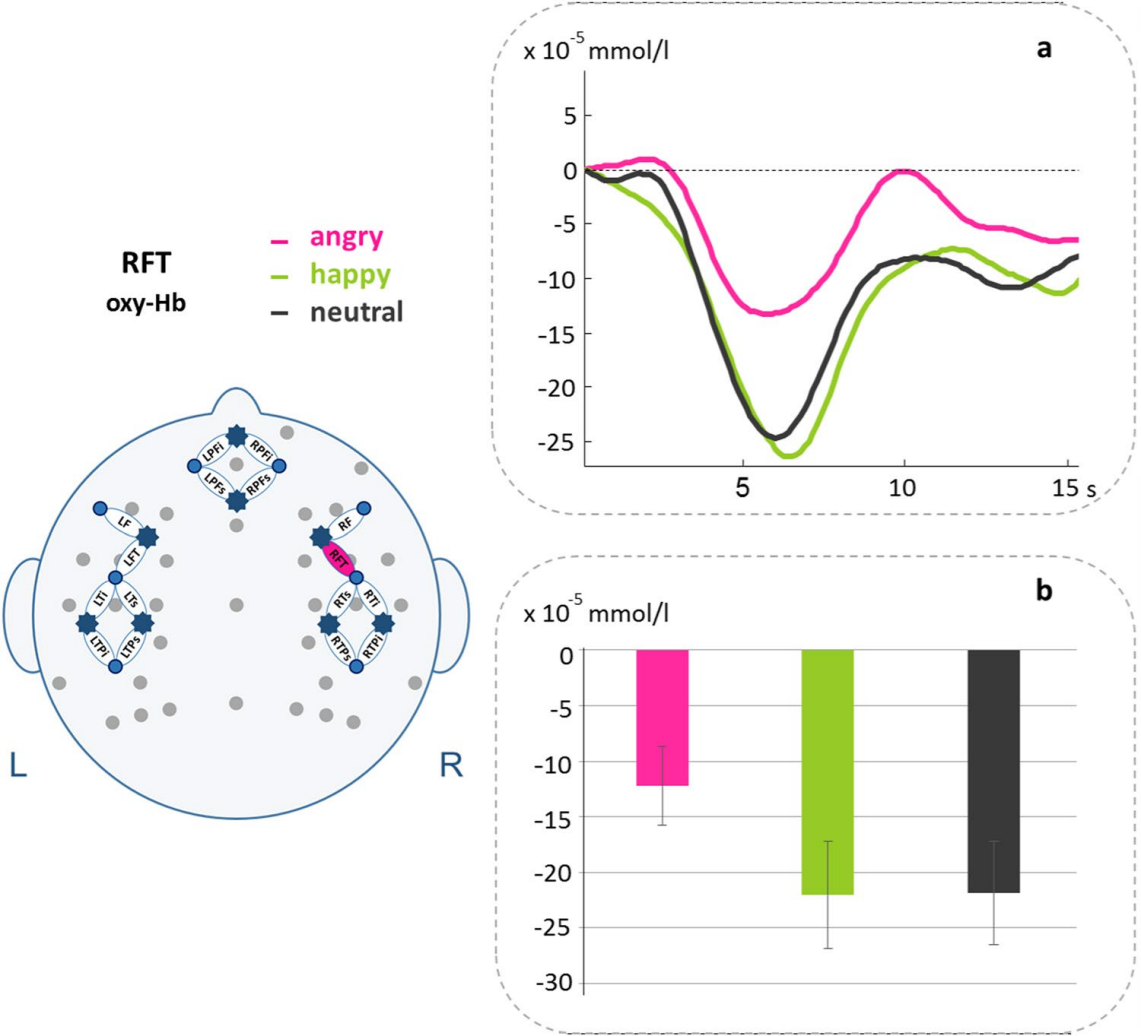
fNIRS results. Time courses (**a**) and bar charts of Beta values including SEMs (**b**) for [oxy-Hb] for angry compared to happy and neutral pseudowords on right fronto-temporal channel RFT.

A four-way ANOVA (Condition × Halves × Hemisphere × Region) was performed for [oxy-Hb] and [deoxy-Hb] separately. This ANOVA did not result in any significant main or interaction effects. In order to get some insights in the underlying neural mechanisms, we then performed a one-way ANOVA with the factor Condition (angry, happy, neutral) on each channel. No significant results were obtained for [deoxy-Hb], but for [oxy-Hb] a significant main effect for Condition was found on right fronto-temporal channel RFT [*F*(2,1000) = 3.647, *p* = 0.039]. Post-hoc *t*-tests revealed increased activations for angry compared to both happy [*t*(43) = 2.043, *p* = 0.047] and neutral [*t*(43) = 2.206, *p* = 0.033] prosody on this channel (cf. Figure 3), however they did not survive FDR correction.

### Post-hoc correlational analysis

A post-hoc correlational analysis was applied to reveal possible relations between EEG and fNIRS results. Both the fNIRS and the EEG revealed one corresponding emotional prosody processing effect, that is, increased activation for angry compared to happy prosody. In the fNIRS, this effect was found on right fronto-temporal channel RFT with increased [oxy-Hb] levels for angry compared to happy prosody. In the EEG, increased negativity for angry compared to happy prosody was found in time window 500–550 ms on frontal, centro-parietal, and parietal superior regions as well as midline electrodes Fz and Cz.

Differences (Δ) between angry and happy prosody for ERP voltage and fNIRS [oxy-Hb] values entered one-tailed Pearson’s correlation analyses. We performed this procedure for all above mentioned significant regions and electrodes in the EEG as well as on channel RFT in the fNIRS.

**Figure 4:**
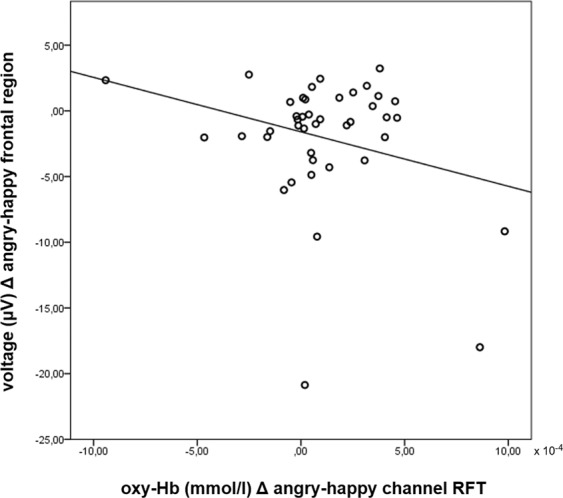
Scatter plot of EEG voltage and fNIRS [oxy-Hb] differences (Δ) between angry and happy prosody showing a significant negative correlation between voltage change on the frontal region and change in [oxy-Hb] on channel RFT.

Results revealed a significant correlation between the increase of [oxy-Hb] on channel RFT and the increase of negativity in the EEG on the frontal region (*r*^2^ = −0.270, *p* = 0.042) (Fig. 4). No significant correlations were found for centro-parietal (*r*^2^ = −0.015, *p* = 0.462) and parietal superior regions (*r*^2^ = −0.068, *p* = 0.334) as well as for midline electrodes Fz (*r*^2^ = −0.200, *p* = 0.102) and Cz (*r*^2^ = −0.055, *p* = 0.365).

## Discussion

The present study aimed at investigating neural mechanisms underlying implicit emotional prosody processing by acoustically presenting single pseudowords conforming to happy, angry, and neutral prosody patterns. Electrophysiological and hemodynamic responses were assessed by a simultaneous multi-methodological approach using electroencephalography (EEG) and functional near-infrared spectroscopy (fNIRS).

### ERPs

Event-related brain potentials (ERPs) revealed significant differences between the three prosodic categories in four different time windows. In time window 250–350 ms, a tendency of larger positivities for happy than angry prosody was found on bilateral frontal and centro-parietal ROIs with waveforms of amplitudes reflecting a P200 component. In research on emotional prosody processing, such fronto-centrally distributed positivities were previously reported and seem to be part of initial emotional encoding and valence identification mechanisms as well as emotional arousal processing^43^. Increased P200 components for happy compared to angry stimuli therefore indicate an elevated initial arousal for happy pseudowords, which is not surprising as happy prosody is defined by higher pitch and richer melodic contour. Increased P200 components for happy prosody compared to other emotional patterns have also been found in previous studies^15,54^.

Interestingly, the present study further revealed a broadly distributed increase in P200 components for neutral compared to both angry (on frontal, fronto-temporal, centro-parietal and parietal ROIs as well as midline electrodes) and happy prosody (on midline electrodes Cz and Pz). Modulations in P200 between emotional and neutral input are in line with the literature. However, previous research depicts that emotional stimuli usually elicit larger positivities than neutral stimuli. This is true for studies with familiar real words^14^, pseudosentences^54^, and nonverbal vocalizations^55^. An increased P200 for neutral pseudowords in the context of emotional prosody does not seem to fit the above-mentioned model of amplitudes increasing with emotional arousal as neutral pseudowords should not provide any affective content.

The P200 has also been related to processes involving lexical access and linguistic memory^56–58^. Therefore, we propose that increased P200 components for neutral stimuli might reflect a pure cognitive mechanism of comparing the acoustic input with representations in linguistic memory, as pseudowords used in the present study conformed to foreign phonotactic rules unknown to the subjects. While neutral words detached from emotional cues elicit phonological search mechanisms, additional emotional input (in this case through prosodic contour) therefore seems to cover up such cognitive processes. This assumption is further supported by the fact that some research has found increased P200 components for neutral compared to emotional stimuli when the material consisted of more complex lexical sentences with semantic content (e.g.^54^).

Research on emotional prosody processing indicates that modulations in P200 components predict changes in late positive potentials (LPPs) which are strongly related to more elaborate processing of emotional arousal and emotional relevance^14,43,44,46,47^. This prediction ability also came into appearance in the present study. Time windows 600–700 and 700–900 ms revealed a long-lasting positive going wave for happy prosody compared to both neutral and angry pseudowords. In time window 600–700 ms, this positivity for happy prosody was significant on all lateral ROIs, while the effect got more centro-parietally distributed in time window 700–900 ms indicating that topographical distribution of the LPP might get more condensed as emotional arousal processes develop. When compared to neutral stimuli, often negative as well as positive intonation patterns elicit increased LPPs^15^. Visual inspection of grand averages of the present study revealed slowly increasing positivities also for angry prosody compared to neutral prosody at later time windows. This effect only got significant on electrode Fz (600–700 ms) and all midline electrodes between 700–900 ms which indicates that mechanisms of emotional arousal and relevance were predominantly active when processing happy emotional prosody.

Interestingly, differences between angry and neutral stimuli compared to happy pseudowords were found in time window 500–550 ms reflected by increased negativities for angry and neutral prosody accompanied by a larger positivity for happy pseudowords. Presenting real but semantically neutral verbs to participants, Grossmann *et al*.^11^ also found increased negativities for angry compared to happy voices between 400 and 600 ms of stimulus onset. Authors state that an increased negativity for angry stimuli in this time frame might indicate greater processing effort or control for angry than happy voices and therefore reflects the mechanism of a negativity bias^59^. Overall, the negativity bias is evolutionary explainable as negative stimuli often go in line with a potential threat and has been described as a fast involuntary response that initiates automatically when subjects are confronted with aversive stimuli in the visual domain^60^. Researchers still debate to what extent such responses are independent of focused (visual) attention to the stimulus^61–63^. Finding such an effect during implicit emotional prosody processing, however, strengthens the assumption that a negativity bias – at least in the auditory domain – functions automatically as no explicit task was given in the present study that could have drawn attention to a specific emotional prosodic category. Therefore, early negative ERPs could reflect a natural protection mechanism in form of an emotional valence identification and threat evaluation process implicitly focusing on aversive stimuli at first.

Unfortunately, in the study by Grossmann *et al*.^11^, emotional voices where not directly compared to neutral stimuli and interpretations of why neutral pseudowords in the present study elicit similar negativities as angry pseudowords remain speculative. Please note that differences between neutral and happy stimuli might be driven by a larger positivity found for happy pseudowords rather than increased negativities for neutral stimuli at least at more posterior electrode sites. Nevertheless, we propose that larger negativities found for angry and neutral prosody compared to happy stimuli might both reflect mechanisms of increased processing effort as they occur in the same time window. However, topographical distribution of effects clearly differed, possibly reflecting diverse driving forces. Differences between neutral and happy prosody were located mainly on frontal and fronto-temporal regions, while increased negativities for angry prosody comprised frontal, centro-parietal and parietal brain areas. We suggest that angry prosodic cues are clearly categorized as threatening, therefore leading to increased emotional processes (i.e., threat evaluation). Neutral pseudowords carrying no additional information whatsoever, neither emotionally nor semantically, might underlie increased processing effort due to an attempt of emotional valence identification as well, as the brain does not know how to deal with them at first since they do not fit an emotional category. We are aware of the fact that this last mentioned interpretation remains speculative and necessitates further research.

Overall, EEG results imply that when processing emotional pseudowords implicitly, angry stimuli still elicit LPPs after a short threat evaluation as they deliver more emotional information than neutral pseudowords, but LPPs are reduced compared to happy stimuli as emotional relevance is minimal for the current situation. Neutral pseudowords underlie an initial form of valence identification effort as well, but are not followed by an LPP because emotional relevance and arousal are non-existent. In addition, happy stimuli are not in need of additional valence identification processes as they are easily categorizable as positive and non-threatening due to their unique prosodic contour (hence no negative amplitudes), but still more arousing compared to the other speech stimuli indicated by very pronounced LPPs. Such ERP sequences during emotional prosody processing might only come into appearance when the speech input is short (i.e., single words), lacks additional semantic information, and no explicit tasks are given that could potentially alter neural responses.

### fNIRS

Neuroimaging studies often fail to find differences between stimuli with positive or negative valence and mostly reveal differences between emotional and neutral conditions (e.g.^40–42^). We found a significant effect for angry compared to both happy and neutral prosody on a right frontal NIRS channel (RFT). Interestingly, the larger activation for angry pseudowords (i.e., increase in [oxy-Hb]) seems to be driven by a larger decrease in [oxy-Hb] for happy and neutral stimuli. Such inverse hemodynamic responses (i.e., an increase in [deoxy-Hb] or a decrease in [oxy-Hb]) were found due to complexity of stimuli^64,65^. This seems to be a potentially fitting explanation for our results as pseudowords consisted of Slovak phonotactic rules completely new and unknown to the monolingually German raised participants and could therefore indeed be interpreted as more complex stimuli. A further, more methodologically oriented explanation is the application of inappropriate baselines during data averaging^66^, especially in event-related designs with short stimuli as applied in our study. However, a GLM approach can handle with such phenomena^65,66^ and was already successfully adopted by our research group^67^. The “neuronal inhibition” hypothesis^68,69^ and the “vascular stealing” hypothesis^66,70^ provide another explanation for inverted responses by attributing a decrease in activation to stimulus repetition and more importantly to an attentional shift. More attention to certain stimuli leads to an increased activation in involved areas and a deactivation in uninvolved neighboring areas. Assuming such an explanation for the inverted response in our study is rather speculative as we find the activation for all emotions in the same brain area. However, this could mean that angry stimuli show stronger activations in a right frontal brain region due to neural deactivation during presentation of happy and neutral pseudowords. It appears clear that more physiological and cognitive studies directly addressing the issue of inverted hemodynamic signals are needed.

Channel RFT comprises adjacent brain regions of both the right inferior frontal cortex (IFC) and the right medial frontal cortex (MFC)^71^. Activations in the right hemisphere during emotional processing are common in the literature^26,32^. In a recent network model for emotional processing, both regions have been related to evaluation processes of the emotional stimulus. In particular, the IFC has been assigned to cognitive evaluation processes of emotional information sent from the superior temporal cortex (STC) while the MFC seems to be predominantly involved during emotion appraisal, evaluation and regulation processes of information sent from the amygdala^33^. To go even further, both the right MFC and the right IFC seem to be involved in the active judgment of emotional prosody^72–74^. Imaging studies on emotional prosody often fail to find differences in activation between positive and negative intonation patterns^42^. Studies that find differences between emotional categories are difficult to compare due to variations of study design and materials used, but show a tendency of increased activations for happy prosody in the left inferior frontal gyrus and bilateral activations or even more right hemispheric activation in STC, orbitofrontal cortex (OFC), and MFC for angry prosody when compared to neutral prosody^38,42,74^. Finding activations for angry compared to happy and neutral prosody in right IFC and MFC therefore does not contradict previous research. Angry stimuli seem to elicit emotion evaluation processes in the fNIRS that go in line with the negativity effect between 500 and 550 ms found in the EEG indicating an evolutionary explainable negativity bias. This was supported by a post-hoc correlational analysis revealing a significant correlation between an increase in ERP negativity as well as an increase in [oxy-Hb] for angry compared to happy prosody.

Unfortunately however, our found fNIRS effect is rather small as it did not survive FDR correction. This could be due to the fact that the fNIRS mostly measures cortical layers of the brain, not reaching deeper structures like the amygdala, which is known to be predominantly involved in emotional processing in general^35^, but also emotional prosody processing in particular^36^. Nevertheless, fMRI studies showed that the cortex is indeed also involved when it comes to emotional (prosody) processing^13,33,37^ and effects were also found in previous fNIRS studies^20,40–42^. However, also in those fNIRS studies, effects are sometimes reported as mere tendencies as statistical analyses reveal only marginally significant results. Notably this is the case even when applying fNIRS as a single method - which bears the potential to assess brain areas in a more fine-grained manner using more channels than we were able during our multi-methodological approach due to the limited space in the EEG cap. fNIRS might therefore be better indicated for study designs investigating processes predominantly comprising cortical structures like linguistic mechanisms, where it has been successfully applied multiple times^10^.

### Conclusions

Overall, results of the present study indicate that positive and negative emotions can be discriminated from each other as well as from neutral prosody on a neural level even when the speech input does not provide any semantic content and no explicit discrimination task is given. Implicit processing of emotional prosody seems to be predominantly driven by mechanisms of emotional and phonological encoding (i.e., P200) as well as emotional valence identification (i.e., increased negativity) at first, followed by processes of emotional arousal and relevance (i.e., LPP) of the respective input. In the EEG, we found larger LPPs for emotional (angry and happy) prosody compared to neutral prosody, most pronounced for happy prosody due to specific intonation patterns (i.e., higher pitch, richer melodic contour etc.) eliciting increased emotional arousal. At earlier processing stages, however, we found larger negativities for angry prosody in the EEG in line with increased activations in the fNIRS indicating an implicit focus to angry and therefore threatening stimuli reflecting a threat evaluation mechanism in the sense of a negativity bias. The identification of several emotional processing steps in healthy subjects is crucial for the investigation of psychiatric patient groups (e.g., suffering from anxiety, post-traumatic stress disorder, depression) exhibiting emotional regulation deficits.

Most noteworthy, this study emphasizes the benefit of a simultaneous EEG/fNIRS approach, as both neuroscientific methods bear the potential to complement each other in getting substantial and profound insights into brain mechanisms underlying specific processes. Concordant results between both methods confirm the assumption that electrophysiological and vascular signals are at least partially interconnected.

## Material and Methods

### Participants

**Figure 5:**
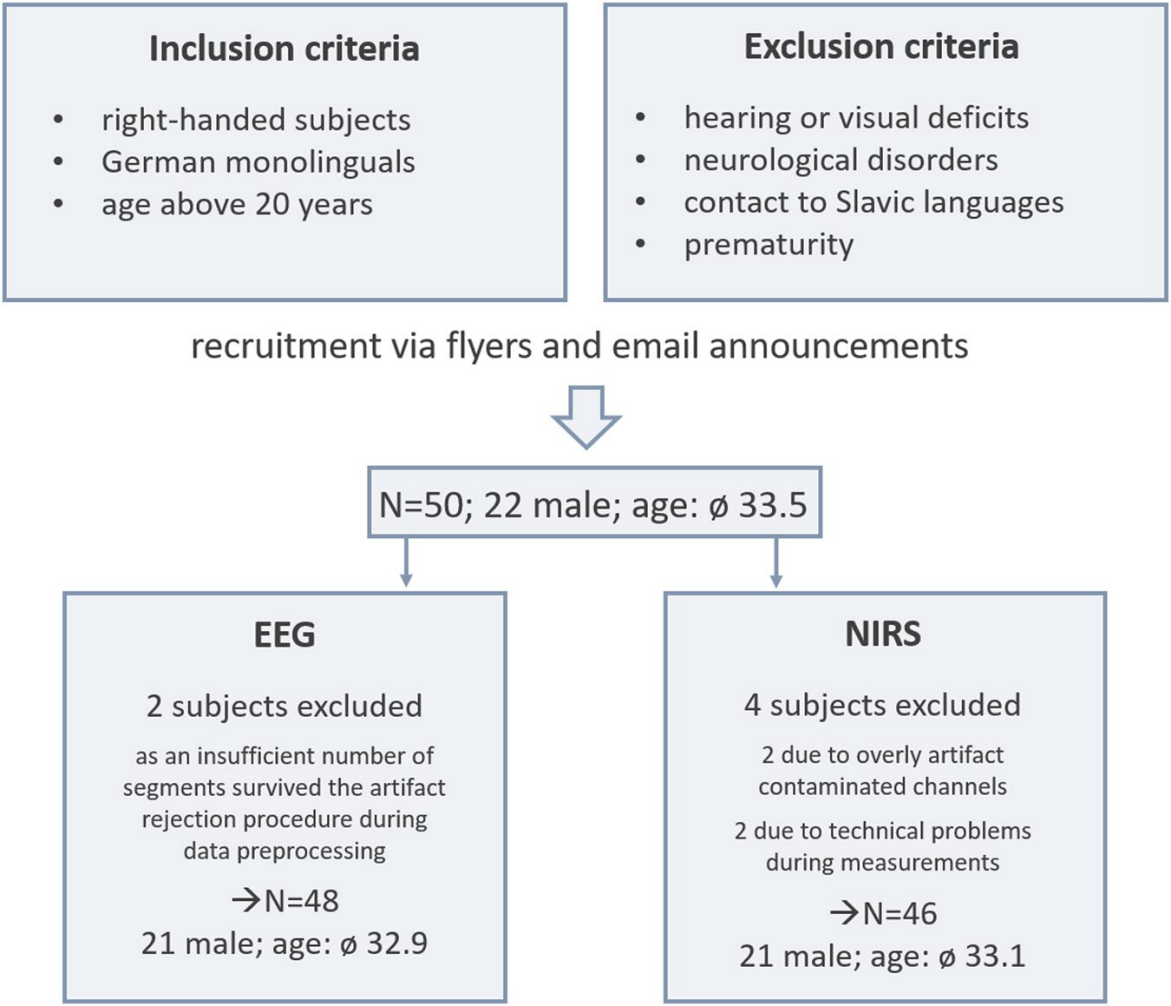
Overview of the participant screening and inclusion process.

In total, fifty healthy adults (22 male) participated in the present study. Due to overly contaminated EEG channels during artifact rejection, two subjects had to be excluded from EEG analyses. Data of four subjects (different from those excluded in the EEG) had to be excluded from fNIRS analyses due to technical problems during measurements. All 50 participants were 33.48 years old on average (range: 19–51 years). All were healthy, had no neurological disorders, did not suffer from hearing or visual impairments and no prematurely born subjects were included in the study (please refer to Fig. 5 for the participant inclusion process).

As the present investigation contained language-related stimuli, linguistic background of all subjects was investigated. Some studies showed that being raised bilingually from birth alters brain development and especially impacts cognitive abilities relevant during processing of linguistic stimuli (e.g.^75^). All participants were raised monolingually with German as their native language. Importantly, subjects had no knowledge in Slavic languages. This was strictly controlled for as the stimulus material included linguistic cues of the Slovak language which should be unfamiliar to the participants.

We assessed handedness in all subjects using the Oldfield Handedness Inventory^76^. All participants were right-handed with a mean score of 84.44 (range: 41.7–100). Level of education was rather high in the present study (university degree: n = 34; high school: n = 10; general education: n = 6).

### Material

The aim of the present study was to investigate implicit processing of pure emotional prosody. Therefore, 30 pseudowords without any semantic content were constructed, as real words with meaning per se could confound emotional processing at the individual subject level. All pseudowords consisted of CCVCV (consonant-consonant-vowel-consonant-vowel) combinations. Onset consonant clusters conformed to phonotactic rules of the Slovak language, which belongs to the Slavic languages. The Slovak language has proven to be very suitable for language study designs with German native speakers since it provides a greater variability of consonant combinations in word onsets than the German language^77,78^. Thus, the linguistic rules of pseudowords were unfamiliar to all participants and hereby we were able to minimize the risk of an impact of individual experiences with the respective speech input.

For all 30 pseudowords, the following onset consonant clusters typical for Slavic languages and non-existent in German were chosen: /bd/, /dw/, /tm/, /fp/, /fn/. Six bisyllabic pseudowords were formed per onset cluster (e.g., fpogo, bdafa, tmipi), while additionally ensuring that the frequency of vowels and consonants in all pseudowords was equally distributed.

The voice recordings took place in an anechoic chamber (Laboratory for Psychoacoustics at the Department of Hearing, Speech, and Voice Disorders of the Medical University of Innsbruck) and were performed by a female speech scientist. All 30 pseudowords were spoken in based on imagined interactions with a conversation partner, each once with neutral, happy, and angry intonation patterns. Additionally, all pseudowords were spoken with a trochee stress pattern on the first syllable corresponding to the most frequent stress pattern in German and thus, not further introducing another foreign language factor to the material. All stimuli were recorded at 44 kHz and 16 bit sampling rate. Afterwards, the acoustic stimuli were edited using the editing programme *Audacity* (www.audacityteam.org). This procedure predominantly included cutting, inserting a short silence period of 30 ms at the onset and offset of each pseudoword, and normalizing loudness. Statistical analyses revealed that all words of different emotional categories did not differ in volume [*F*(2,58) = 0.444, *p* = 0.574] and mean duration [*F*(2,58) = 0.724, *p* = 0.402], but in mean pitch [*F*(2,58) = 634.406, *p* < 0.001]. Happy pseudowords were significantly higher in mean pitch than both neutral [*t*(29) = 29.431, *p* < 0.001] and angry [*t*(29) = −24.398, *p* < 0.001] pseudowords. In order to guarantee that all prosodic styles indeed reflected the respective emotional prosody, participants of the present study were asked to rate all stimuli in a separate behavioral experiment. In total, 77% of angry, 90% of happy, and 96% of neutral stimuli were identified correctly. Participants were also asked to rate perceived threat from all pseudowords to measure emotional content of the speech input on a 1 to 8 point likert scale. As expected, angry pseudowords elicited the largest amount of perceived threat (M = 4.72) when compared to happy (M = 1.88) and neutral (M = 2.34) pseudowords.

### Procedure

Neural activity was assessed simultaneously by means of electroencephalography (EEG) and functional near-infrared spectroscopy (fNIRS). The former method excellently tracks fast processing mechanisms in the millisecond range, whereas the latter provides a good spatial resolution indicating the brain areas recruited. This multi-methodological approach has proven to be highly beneficial for investigating acoustic stimuli, as both methods are soundless, do not interfere with each other, and provide a quite natural setting^10^.

The study was approved by the ethical committee of the Medical University of Innsbruck (Austria). The experiment was carried out in accordance with relevant guidelines and regulations (Declaration of Helsinki) and written informed consent was obtained from all participants prior to measurements. During the experiments, subjects sat on a chair 1 m in front of a computer monitor. To enable a simultaneous measurement of both methods, subjects wore elastic EEG caps (EasyCap GmbH, Herrsching, Germany) in which both EEG electrodes as well as fNIRS optodes were integrated. Pseudowords were acoustically presented via loudspeakers at an intensity of approximately 70 dB. As we wanted to investigate implicit emotional processing, no specific task was applied and participants listened passively to the stimuli.

**Figure 6:**
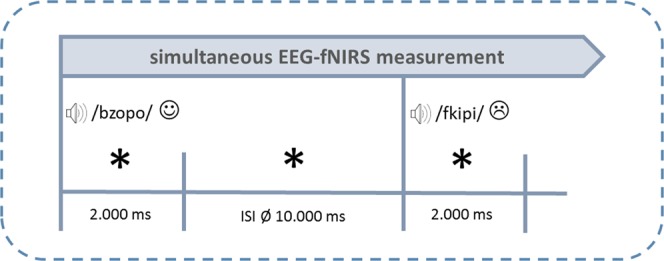
Experimental design.

The presentation of each pseudoword lasted 2 s followed by a variable inter-stimulus-interval (ISI) (mean duration: 10 s, range: 6–14 s). By introducing this variable ISI, the experimental design was adjusted to the requirements of the rather slow hemodynamic response measured by the fNIRS. Usually, vascular responses reach their maximum at around 5 s after stimulus presentation with the activation returning to baseline after 15–20 s^79^. Therefore, variable ISIs prevent hemodynamic responses from overlapping systematically. Furthermore, all pseudowords were presented in a pseudorandomized manner with the following criteria: (1) maximally three consecutive pseudowords of one and the same emotional category, (2) at least three different pseudowords between words with the same onset clusters, and (3) an equal distribution of all emotions between experiment halves. In total, 4 pseudorandomization versions were used throughout the measurements. The experiment lasted 18 minutes. Please refer to Fig. 6 for a depiction of the experimental design.

### EEG recordings

**Figure 7:**
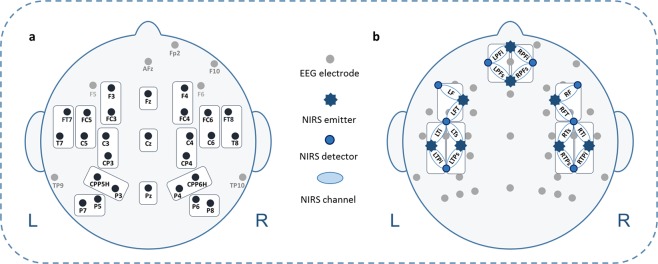
Simultaneous EEG electrode and fNIRS channel placement. (**a)** EEG electrode configuration including regions of interest (ROIs). (**b)** fNIRS channel arrangement including ROIs: stars indicate 8 fNIRS light emitters; dots indicate 8 fNIRS detectors; ellipses indicate fNIRS channels; channels cover prefrontal inferior (PFi), prefrontal superior (PFs), frontal (F), fronto-temporal (FT), temporal inferior (Ti), temporal superior (Ts), temporo-parietal inferior (TPi), and temporo-parietal superior (TPs) brain regions, for both hemispheres respectively. Additionally, all 8 left-hemispheric fNIRS channels are marked with an L; all 8 right-hemispheric fNIRS channels are marked with an R.

EEG was recorded from 32 AgAgCl active electrodes (BrainProducts GmbH, Gilching, Germany) placed into an elastic EEG cap at the following positions of the 10–20 system^80^: F5, F3, FT7, FC5, FC3, T7, C5, C3, CP3, CPP5H, P7, P5, P3, F4, F6, FC4, FC6, FT8, C4, C6, T8, CP4, CPP6H, P4, P6, P8, Fz, Pz, and Cz (cf. Figure 7). Vertical and horizontal electrooculogram were recorded above and next to the right eye with electrodes FP2 and F10. An electrode (TP9) at the left mastoid served as online reference, while an electrode at the right mastoid (TP10) was recorded for further re-referencing during offline analyses. Position AFz served as ground electrode. Electrode impedance was kept below 10 kΩ (actiCAP Control, Brain Products GmbH, Gilching, Germany). The EEG signal was measured by means of BrainVision Recorder (Brain Products GmbH, Gilching, Germany) software with a sampling frequency of 1000 Hz (amplified between 0.016–450 Hz) and filtered before digitalization by means of the analog/digital converter with an upper cut-off of 450 Hz (24 db/oct) to prevent aliasing.

### fNIRS recordings

Vascular changes were measured by means of functional near-infrared spectroscopy. With this method, concentration changes of both oxygenated [oxy-Hb] and deoxygenated hemoglobin [deoxy-Hb] in cortical brain areas can be assessed by emitting light in the near-infrared spectrum to the biological tissue. Calculations of concentration changes in both hemoglobins are based on a modified Beer-Lambert law^81^. The physiological basis of fNIRS is the neurovascular coupling: an increased activation in a brain region leads to several vascular and metabolic changes. It is known that vasodilation leads to a local increase in blood volume demanding more oxygen and glucose, which in turn leads to an increase in regional cerebral blood flow and an increase in regional blood flow velocity^82^. In consequence, the color of the blood changes. The blood flow increase overcompensates oxygen consumption and elicits a focal hyperoxygenation resulting in an increase in oxygenated hemoglobin [oxy-Hb] as well as a decrease in deoxygenated hemoglobin [deoxy-Hb]^83^. [Deoxy-Hb] is inversely correlated to the BOLD signal measured by functional magnetic resonance imaging (fMRI) (for more details see^84^).

The used fNIRS system (NIRScout, NIRx Medizintechnik GmbH, Berlin) sends wavelengths at 760 and 850 nm in a cw-mode and recorded data at a sampling rate of 7.81 Hz. In total, eight light emitters and eight light detectors were used to assess activations over bilateral fronto-temporo-parietal brain areas. Interoptode distance was 3.5 cm. This emitter-detector configuration allowed the assessment of 8 channels per hemisphere, covering prefrontal inferior (PFi), prefrontal superior (PFs), frontal (F), fronto-temporal (FT), temporal inferior (Ti), temporal superior (Ts), temporo-parietal inferior (TPi), and temporo-parietal superior (TPs) brain regions. Positioning of fNIRS light emitters and detectors was based on the standard EEG 10–20 electrode positioning system^80,85^. Recent studies in adults^71^ used this electrode positioning to project to underlying anatomical structures in order to provide a better mapping of signals assessed from the scalp.

### Data analyses

#### EEG data

EEG data was filtered offline with a 30 Hz low pass Butterworth zero phase filter (high cutoff: 30 Hz; slope: 12 dB/oct). Data was then segmented from −200 ms to 1500 ms with 0 ms representing the time point of the pseudoword onset. An ocular correction based on the Gratton & Coles algorithm^86^ was applied to correct vertical eye movement artifacts. Overly contaminated channels were rejected manually from each segment by inspecting each segment visually for artifacts. Only subjects in whom at least 2/3 of all segments per condition (angry vs. happy vs. neutral) in at least 15 of all 29 scalp electrodes survived this procedure were included in the final analyses. This criterion applied to 48 of all 50 subjects. In the next steps, data was re-referenced to averaged mastoids (TP9, TP10) and a pre-stimulus baseline of 200 ms was applied.

Event-related brain potentials (ERPs) were extracted by averaging the segments for each subject and each condition (angry prosody, happy prosody, neutral prosody). In addition, a 50-ms-analysis was performed in order to select the time windows for final statistical analyses. This analysis included ANOVAs on each electrode in consecutive 50 ms steps between 100 and 1500 ms with the factor Condition for experimental halves separately. We decided to include the factor halves into all statistical analyses, as we wanted to control our results for potential habituation/repetition effects over the course of the experiment. Results from the 50-ms-analysis as well as visual inspection of the grand averages revealed 100–150 ms, 250–350 ms, 500–550 ms, 600–700 ms, and 700–900 ms to be the time windows indicating differences between conditions, which were therefore were selected to perform further statistical analyses on.

Since the topographical localization of the EEG is only rough, we decided to perform final statistical analyses on 12 regions of interest (ROIs). The following lateral ROIs were included: left frontal (F3, FC3), right frontal (F4, FC4), left fronto-temporal superior (FC5, C5), right fronto-temporal superior (FC6, C6) left fronto-temporal inferior (FT7, T7), right fronto-temporal inferior (FT8, T8), left centro-parietal (C3, CP3), right centro-parietal (C4, CP4), left parietal superior (CPP5H, P3), right parietal superior (CPP6H, P4), left parietal inferior (P5, P7), and right parietal inferior (P6, P8) (cf. Figure 7). Midline electrodes (Fz, Cz, Pz) were analyzed separately. We then performed a four-way repeated-measures ANOVA for lateral ROIs with the within-subject factors Condition (angry vs. happy vs. neutral), Halves (first experiment half vs. second experiment half), Hemisphere (left hemisphere vs. right hemisphere), and Region (frontal vs. fronto-temporal superior vs. fronto-temporal inferior vs. centro-parietal vs. parietal superior vs. parietal inferior) for all time windows, respectively. For midline electrodes, ANOVAs with the factors Condition, Halves, and Electrode (Fz vs. Cz vs. Pz) were performed. Whenever a main effect of Condition or an interaction with Condition reached significance, post-hoc *t*-tests were subsequently performed. Significance level was set at *p* ≤ 0.050 and adjusted with the False Discovery Rate (FDR) procedure^87^. Corrected significance according to Greenhouse-Geißer^88^ was applied whenever the degrees of freedom exceeded 1.

#### fNIRS data

In order to analyze concentration changes (mmol/l) of [oxy-Hb] and [deoxy-Hb], the collected reflected light was transformed by means of the modified Beer-Lambert function^81^. Exclusion of artifacts (e.g., abrupt changes, mainly due to head movements) of each participant was performed manually. Artifacts were removed by a linear spline interpolation approach which has been frequently used (e.g.^67^,) and is preferred compared to methods rejecting artifact-contaminated segments^89,90^ as it allows keeping a large amount of data^89^. A 0.4 Hz low pass filter (Butterworth, third order) was applied to attenuate high-frequency artifacts mainly arising from heartbeat. We applied a general-linear-model (GLM) instead of averaging data as it can better describe the complex hemodynamic response function (hrf). Furthermore, results are more comparable to existing fMRI data where GLM usage is standard procedure. A GLM allows better handling of partial overlap of hrfs which occurs due to the relatively short stimulations and ISIs as part of an event-related design (e.g.^65^). For the GLM, data were correlated with a predictor generated by convolving the boxcar function of the stimulus design including 6 different conditions (angry first experiment half, angry second experiment half, happy first experiment half, happy second experiment half, neutral first experiment half, neutral second experiment half) with the canonical hrf^91^. During this modelling, a stimulation period of 2 s (i.e., on-condition) and a resting period (i.e., off-condition; silence) resulting from ISIs was assumed and a high-pass filter of 20 s to remove drifts and slow fluctuations was applied (for similar procedures please see^67,92^).

Statistical analyses were performed on the beta values of both [oxy-Hb] and [deoxy-Hb] provided by the GLM. Please note that both a decrease in [deoxy-Hb] as well as an increase in [oxy-Hb] are considered as reflections of increased activation, thus we report both hemoglobins separately^84,93^. Every two adjacent channels were then merged resulting in the following 4 regions of interest (ROIs) (left (L) and right (R) hemisphere respectively): prefrontal (LPFi, LPFs, RPFi, and RPFs), fronto-temporal (LF, LFT, RF, and RFT), temporo-parietal superior (LTs, LTPs, RTs, and RTPs), and temporo-parietal inferior (LTi, LTPi, RTi, and RTPi) (cf. Figure 7). We performed a four-way repeated-measures ANOVA with the within-subject factors Condition (angry vs. happy vs. and neutral), Halves (first experiment half vs. second experiment half), Hemisphere (left hemisphere vs. right hemisphere), and region (frontal vs. fronto-temporal vs. temporo-parietal superior vs. temporo-parietal inferior) for [oxy-Hb] and [deoxy-Hb], separately. Whenever a main effect of Condition or an interaction with Condition reached significance, post-hoc *t*-tests were subsequently performed. Significance level was set at *p* ≤ 0.050 and adjusted with the False Discovery Rate (FDR) procedure^87^.

### Correlational analyses between EEG and fNIRS signals

In case of corresponding findings in EEG and fNIRS as single methods, we perform correlation analyses to verify whether electrophysiological and vascular signals show the same underlying processes. For this purpose, differences (Δ) between relevant prosody effects for ERP voltage and fNIRS Beta-values will be submitted to one-tailed Pearson’s correlation analyses. Similar procedures of correlating electrophysiological and hemodynamic responses have already been successfully adopted by other research groups^51,52^.
